# Pattern of stimulants abuse among adolescents and associated risk

**DOI:** 10.1192/j.eurpsy.2026.10487

**Published:** 2026-07-31

**Authors:** N. Nagy

**Affiliations:** NeuroPsychiatry, Ain Shams University, Cairo, Egypt

## Abstract

**Introduction:**

Rates of stimulant use are rising rapidly among adolescents.Recently,ADHD diagnoses rose twofold, along with a similar increase in the number of prescriptions written for stimulants mainly methylphenidate (Ritalin).Deaths involving cocaine and synthetic opioids increased by 50% among youth aged 15–19. Stimulant use is associated with potential harms. Given by injection, increase the risk for human immunodeficiency virus, hepatitis C virus, skin/soft tissue infections, and endocarditis.

**Objectives:**

This study aimed to find out pattern of stimulants abuse among adolescents and correlation with sociodemographic, clinical characteristics and risk complications.

**Methods:**

Cross-sectional descriptive study was conducted on 165 adolescents aged 12-19 years diagnosed as stimulant use disorder according to Kiddie Schedule for Affective Disorders and Schizophrenia, Present and Lifetime version (K-SADS-PL). Informed written consent was signed from patients and their guardians to be part of the study. Data was collected and statistically analyzed using SPSS version 26.

**Results:**

Mean age for the studied group was 15.11+2.33 years, Differences exist in adolescent stimulant use by age and gender. The mean age of initiation is 17 years for methamphetamine and nonmedical prescription stimulant misuse. 82% were males while high school girls report the highest rates of methamphetamine use associated with sexual practices. 61.5% were studying in high schools and 48.12% of average family income. Psychiatric comorbidities detected by KSADS shows 20% suffering from ADHD, 12.3% psychosis and 33.8% had depression. As regards stimulant use route 80% smoked, 20% as tablets with nobody taking injectable forms. 83.1% were polydrug users for cannabis and opioids. They reported using stimulants to balance out the sedative effects of opioids or to manage withdrawal symptoms.

Parental opioid use showed significant high risk factor for adolescent stimulant use. 77.14 % showed high risk score on health, social and legal problems. Methamphetamine precipitates psychosis and have lethal effects on the cardiovascular system, increasing the risk of fatal cardiac events and heart failure.

38.5 % tried to control and stop intake at least twice. The combination of bupropion and naltrexone, mirtazapine and mood stabilizers demonstrated efficacy in treating methamphetamine use disorder.

**Conclusions:**

Stimulant use disorder, including the use of prescription and illicit stimulants, is on the rise among adolescents, along with the serious consequences, including overdose, other medical harms, psychiatric harms, and long-term sequelae of substance use.

**Disclosure of Interest:**

None Declared

